# Neuropeptide Y1 receptor antagonist but not neuropeptide Y itself increased bone mineral density when locally injected with hyaluronic acid in male Wistar rats

**DOI:** 10.3906/sag-2001-268

**Published:** 2020-08-26

**Authors:** Muhammer Özgür ÇEVİK, Petek KORKUSUZ, Feza KORKUSUZ

**Affiliations:** 1 Department of Medical Genetics, Faculty of Medicine, Adıyaman University, Adıyaman Turkey; 2 Department of Histology and Embryology, Faculty of Medicine, Hacettepe University, Ankara Turkey; 3 Department of Sports Medicine, Faculty of Medicine, Hacettepe University, Ankara Turkey

**Keywords:** NPY1 antagonist, neuropeptide Y, hyaluronic acid, bone mineral density

## Abstract

**Background/aim:**

The nervous system controls bone mass via both the central (CNS) and the peripheral (PNS) nervous systems. Intriguingly, neuropeptide Y (NPY) signaling occurs in both. Less is known on how the PNS stimulated NPY signaling controls bone metabolism. The objective of this study was to evaluate whether NPY or NPY1 receptor antagonist changes local bone mineral density (BMD) when injected into a Wistar rat tibia.

**Materials and methods:**

Tibial intramedullary area of 24 wild type male Wistar rats (average weight = 350 ± 50 g, average age = 4 ± 0.5 months) were injected with NPY (1 × 10^-5^ M and 1 × 10^-6^ M) and NPY1 receptor antagonist (1 × 10^-4^ M) dissolved in hyaluronic acid (HA) separately. Tibiae were collected after one and two weeks. BMD was measured with dual-energy X-ray absorptiometry (DXA) and micro quantitative computer tomography (QCT). Histological changes were analyzed with light microscopy, Goldner's Masson trichrome (MT), and hematoxylin-eosin staining.

**Results:**

According to DXA **,** the mean BMD of NPY dose 1 (1 × 10-5 M) was significantly lower than that of the control (HA applied) group and not significantly but still lower than that of the NPY dose 2 and NPY1 antagonist applied groups. QCT results indicated the same pattern statistically insignificantly in the trabecular area but not in the cortex of the bones. Histologically, only NPY1 antagonist applied tibiae revealed young spongiosis bone trabeculae formed in the borderline of the cortical bones. HA was remarkably biocompatible and late degrading in the tissues.

**Conclusion:**

Local administration of NPY and NPY1 antagonists may hold regulating potential of BMD and bone formation. NPY1 antagonist caused new bone formation in trabecular bone when applied locally. NPY dissolved in HA however can be used to suppress bone formation.

## 1. Introduction

The skeletal system harbors a balance mechanism in which old or damaged bone is periodically removed, and new bone is formed at discrete sites throughout life. This regenerative process of bone remodeling depends on the formative and resorptive activities of specialized bone cells called osteoblasts and osteoclasts [1]. The actions of osteoclasts and osteoblasts are tightly controlled by numerous networks of autocrine, paracrine, or endocrine interactions. These include autocrine and paracrine factors such as cytokines, growth factors, prostaglandins, as well as mechanical stimuli sensed by another specialized cell called osteocytes [2]. 

The nervous system is a major regulator of bone structure and metabolism [3]. Clinical observations showed that patients with neurological disorders exhibited localized osteopenia and bone fragility [4], altered fracture healing [5], and excessive callus formation [6]. Immunohistochemical studies revealed an extensive network of nerve fibers in the vicinity and within the skeleton [7]. Interestingly, phenotyping of the skeletal nerve fibers demonstrated the existence of different signaling molecules, including neuropeptides, neurotransmitters, and neurotrophins [8]. Nervous system elements, including neuropeptides, are therefore always active in the control of bone metabolism via both in the central and peripheral nervous systems.

Neuropeptide Y (NPY) is a 36-amino acid polypeptide that belongs to the NPY related peptides of which consists of the NPY, peptide YY (PYY) and pancreatic polypeptide (PP). NPY family of peptides is all 36-amino acid peptides, and all share a typical hairpin-like loop structure, the PP-fold. This PP-fold structure is essential in regulating their binding to their Y receptors. The target of the NPY family peptides is these Y receptors. The Y receptors are a family of G-protein-coupled receptors with five subtypes: Y1, Y2, Y4, Y5, and Y6 (of which is redundant in humans). The NPY and PYY have similar Y-receptor binding profiles with the highest affinity for the Y2 receptors, followed by Y1, Y5, and the least affinity for Y4 receptors [9]. 

NPY involves various regulation activities related to behavior, food intake, energy by acting as a neuromediator that functions in both central (CNS) and the peripheral nervous system (PNS) [10]. In the CNS, NPY carries our vast number of tasks yet more being discovered [11]. In the PNS, NPY is coreleased with noradrenaline from sympathetic nerves [12]. In addition to nerve cells, other cell types such as adipocytes express both Y1 and Y2 receptors, and osteoblasts express Y1 receptors, indicating the potential for local and even autocrine effects as NPY is expressed in the same cells [13]. 

NPY has intriguing actions on bone metabolism via both CNS and PNS [14]. Notably, the PNS existence of neuropeptide Y (NPY) receptors was demonstrated via histological techniques [15] and subsequently confirmed by RT-PCR [16,17]. However, local effects of NPY and bone tissue interactions remain to be elusive [18] and may provide valuable therapeutic advantages [19,20]. 

The nature of NPY signaling is complex, and so many controversies remain to be cleared [17]. It was shown that ad libitum administration of an NPY1 receptor antagonist increased bone mass in male wild type mice [21], but the study did not differentiate between the antagonist's peripheral and central effects. This distinction is important since a recent NPY conditional knock-out model [22] revealed that the behavior of bone cells differed in vitro from in vivo so that isolated bone marrow stromal cells from these animals had differentiated into osteoblasts in vitro. However, as an organism, male knock-out mice had reduced bone mass and strength in vivo, where the female mice had no change in bone mass compared to wild type [22]. Furthermore, a mice knock-out model that specific overexpression of NPY in only bones displayed increased osteogenesis [23]. Therefore, the local actions of NPY and NPY1 antagonist, as well as HA as a drug carrier substance, are potentially the valuable focus of interest for future therapeutic challenges.

In this study, we assumed that peripheral interactions of NPY and Y1 receptors were responsible for regulating bone mass without CNS regulation. In order to ensure controlled, local delivery of NPY and NPY1 receptor antagonist in the bone medulla, we have chosen HA as the carrier matrix, among other choices [24]. To our knowledge, we used NPY and NPY1 antagonist in a local delivery setting with HA for the first time in the literature.

## 2. Materials and methods

We designed a prospective experimental study. Independent variables were groups (NPY1 = 1 × 10^-5^ M, NPY2 = 1× 10^-6^ M, NPY1 antagonist, HA), and application concentrations were inspired by Sun QQ et al. [25] and Morgan DGA et al. [26]. Time (day one, week one, and week two), and dependent variables were bone mineral BMD as measured by DXA and QCT and histological assessments. DXA scans were carried out in Middle East Technical University Health Center with a Siemens Lunar Machine. Tibiae were put in a water bath, and their anterior surfaces faced up to the vertical to the X-ray source. The bath was filled with distilled water up to 2 cm above the tibial surface. The X-ray beam was composed of 76 kVp and 140 kVp energy levels, both having 2.0 mA and 60 Hz current values. The resolution of DXA was set to the full, small animal setting in which a single scan time extended to 3.5 min. BMD values in g/cm^2^ were recorded for each group.

QCT scans were performed by using Philips Tomoscan Computer Tomography 60/TX3 three times at the Petroleum Research Laboratory (PRL), Petroleum and Natural Gas Engineering Department, Middle East Technical University, Ankara, Turkey. The bone densities and atomic content of the tibia were evaluated. All test specimens were placed inside plastic "falcon" tubes containing isotonic saline solution. Prepared specimens were fixed 60 cm away from the X-ray source on the Tomoscan table. During CT examinations, 576 channel detectors were present to sense 100 kV and 130 kV energy discharges. The time needed to complete one full cross-sectional imaging of the test specimen was 3 s. The distance between the two cross-sectional images was 2 mm. The field of view was 300 mm, and the field length was 4 mm. The current flow through the X-ray source was 250 mA. All captured images were transferred into Intel Pentium 4 powered image analysis software, and calculations were performed by the "CT Density Calculator" software developed by the PRL staff.

For histological analysis, tibia samples collected according to their time points were fixed in 10% formaldehyde and stored until they were evaluated for histological processes. Then, they were decalcified gradually by placing into DeCastro (absolute ethanol, 300 mL), chloraldehyde 50g, DW 670 mL, concentrated nitric acid solution (70%) of 30 mL at room temperature. After decalcification, gradual alcohol treatment was applied for dehydration, and a constant vacuum application was performed. Then the specimens were embedded into paraffin blocks. From paraffin blocks, 4–6 µm thick sections were taken with a rotary microtome (Microm HM 360, Marshall Scientific, Hampton, NH, USA). For histological examination, sections were stained with hematoxylin and eosin and Goldner's Masson trichrome (MT) technique. Stained specimens primarily evaluated from the aspects of tissue repair related to new bone and bone marrow formation. 

All measurements were expressed as means and standard deviations. Data were analyzed using a two-way analysis of variance to assess statistical significance. Significance was accepted as P < 0.05. Independent variables were groups of subjects (four levels; HA, NPY dose 1, NPY dose 2, and NPY-plus inhibitor) and time ( three levels; first day, first week, and second week); dependent variables were DXA measurement, QCT based BMD of cortex, QCT based BMD of medulla, QCT based atomic content of cortex, QCT based atomic content of medulla. SPSS 8.0 program was used for these statistical analyses.

## 3. Results

DXA analysis revealed that the average BMD of NPY applied tibiae decreased when compared to NPY inhibitor or HA (solvent only) applied tibiae. However, this was statistically significant at week one only (P = 0.02 < 0.05) but not at week two (P = 0.86 > 0.05) and there was a group effect between the treatment groups at both weeks [F (3,16) = 6.7, P = 0.004 < 0.05] meaning treatments were different from each other (or had dosage effect). At week one, the BMD of NPY dose 1 (1 × 10-5 M) applied tibiae (X = 0.13 g/cm^2^) was significantly lower than HA applied tibiae (X = 0.18 g/cm^2^, P = 0.002 < 0.05) (Figure 1). Besides, when NPY inhibitor was applied, the decrease in BMD was partially reversed (X = 0.148 g/cm^2^ at week one and X = 0.203 g/cm^2^ at week two) although it was not statistically significant. 

**Figure:**
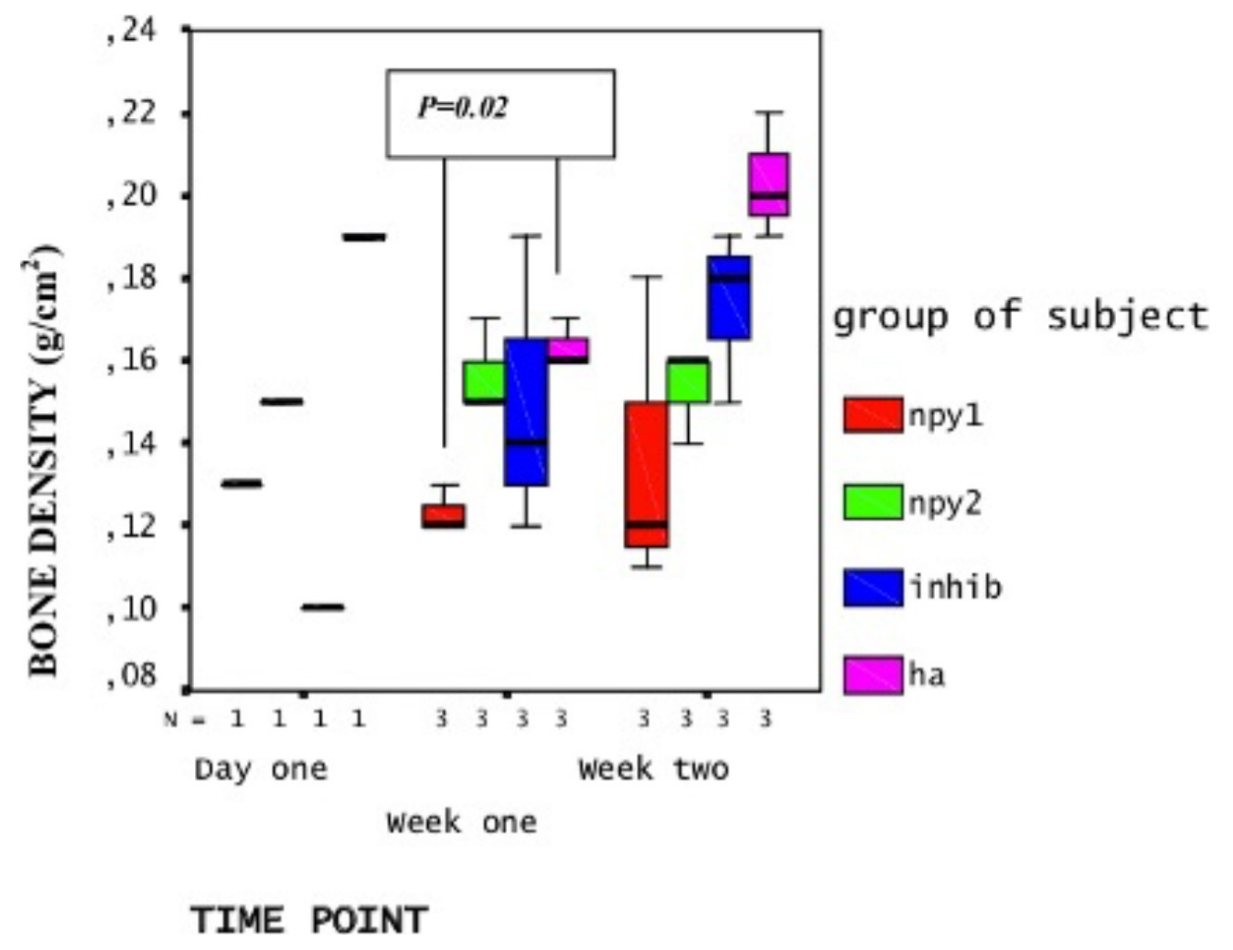
Profile plot of DXA analysis results: Y-axis displays BMD in g/cm^2^, and X-axis displays time point, and N stands for the number of rats analyzed. The group of subjects is displayed in different colors as groups indicated on the right. NPY1 represents the higher dose (1 × 10^-5^ M) and NPY2 represents the lower dose (1 × 10^-6^ M) NPY application. INHIB represents NPY1 receptor antagonist (1 × 10^-4^ M) and HA represents the control (solvent) applied group. Only one rat per group was available at the day 1. At the timepoints week one (7th day) and week two (14th day), each group had three rats.

QCT scans revealed that the change in BMD occurred in the medulla rather than in the cortex region of the tibiae on both time points, although this correlation was statistically not significant (Figures 2a and 2b). That is to say, NPY treatments created a noticeable decrease in average medullary BMD (X = 1.69 g/cm^3^ at week one and X = 1.64 g/cfm^3^ at week two for NPY dose 1; X=1.61 g/cm^3^ at week one and X=1.62 g/cm^3^ at week two for NPY dose 2), and this was reversed on groups treated with NPY1 inhibitor (X = 1.56 g/cm^3^ and X = 1.68 g/cm^3^, respectively) when compared to HA treatment (X = 1.76 and X = 1.65 g/cm^3^, respectively). 

**Figure 2a:**
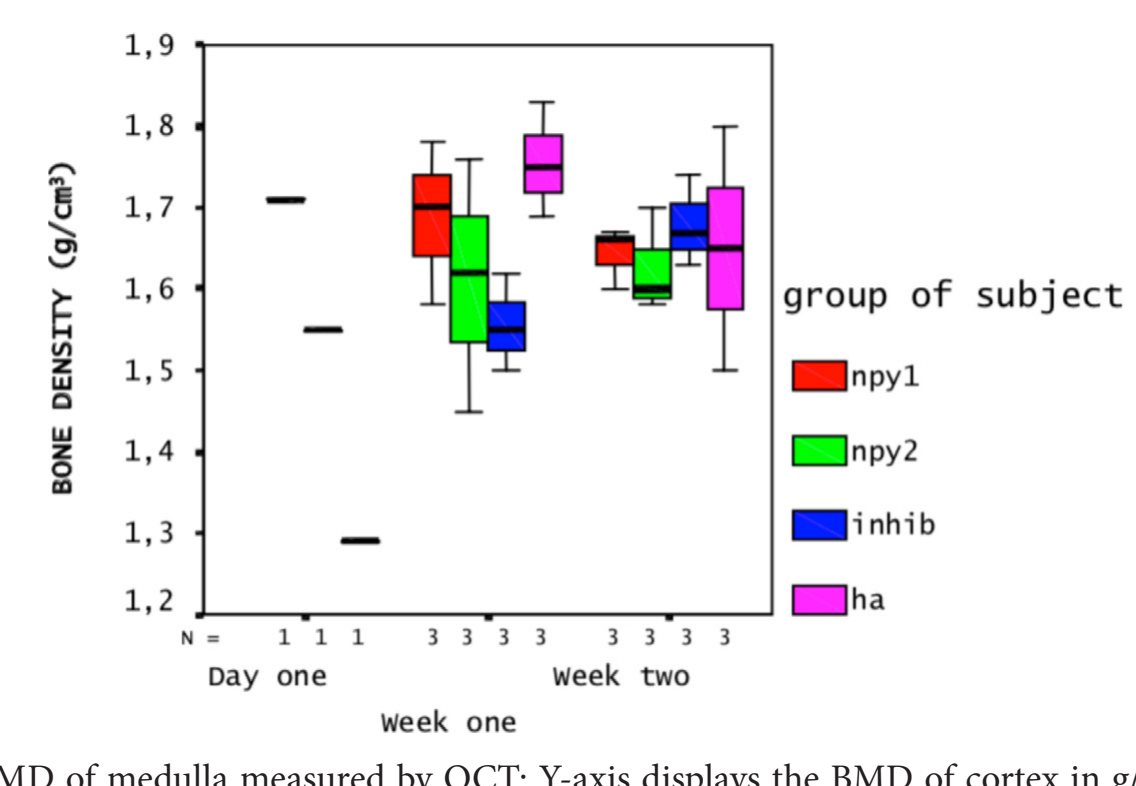
Profile plot of BMD of medulla measured by QCT: Y-axis displays the BMD of cortex in g/cm^3^, the X-axis displays time points and N (number of rats). The group of subjects is displayed in different colors as groups indicated on the right. NPY1 represents the higher dose (1 × 10^-5^ M) and NPY2 represents the lower dose (1 × 10^-6^ M) NPY application. INHIB represents NPY1 receptor antagonist (1 × 10^-4^ M) and HA represents the control (solvent) applied group. Only one rat per group was available at the day 1. At the timepoints week one (7th day) and week two (14th day), each group had three rats.

**Figure 2b:**
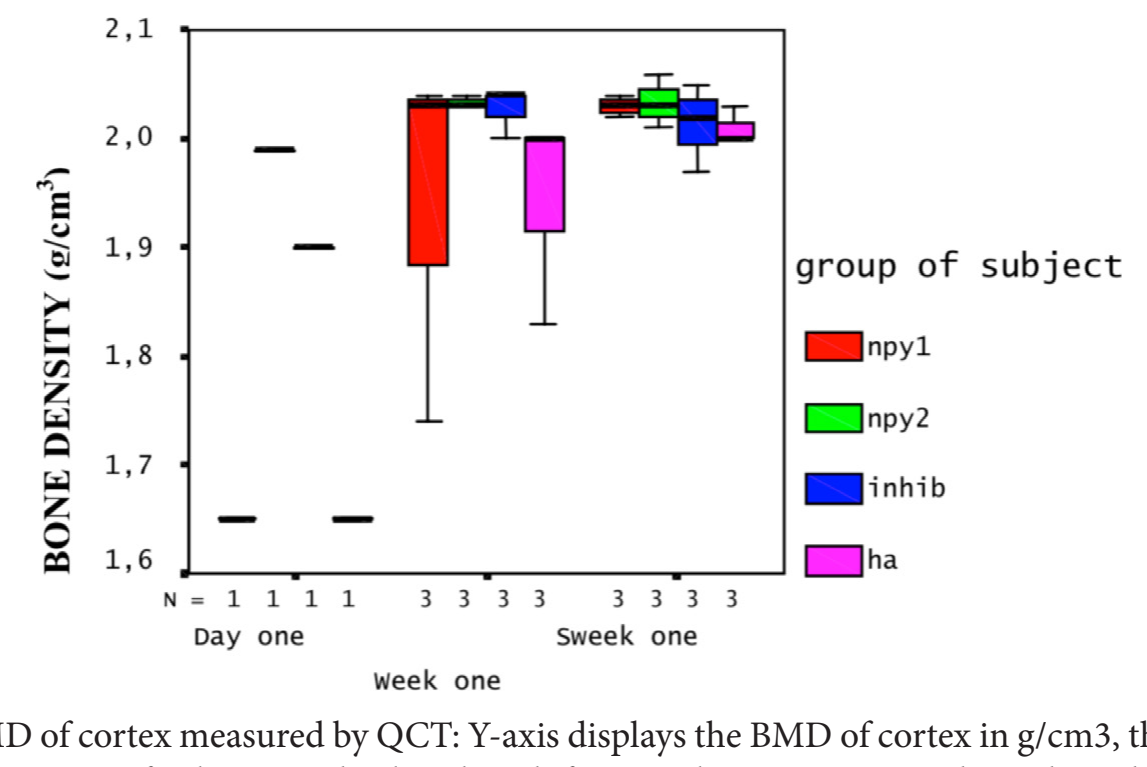
Profile plot of BMD of cortex measured by QCT: Y-axis displays the BMD of cortex in g/cm^3^, the X-axis displays time points and N (number of rats). The group of subjects is displayed in different colors as groups indicated on the right. NPY1 represents the higher dose (1 × 10^-5^ M) and NPY2 represents the lower dose (1 × 10^-6^ M) NPY application. INHIB represents NPY1 receptor antagonist (1 × 10^-4^ M) and HA represents the control (solvent) applied group. Only one rat per group was available at the day 1. At the timepoints week one (7th day) and week two (14th day), each group had three rats.

**Figure 3:**
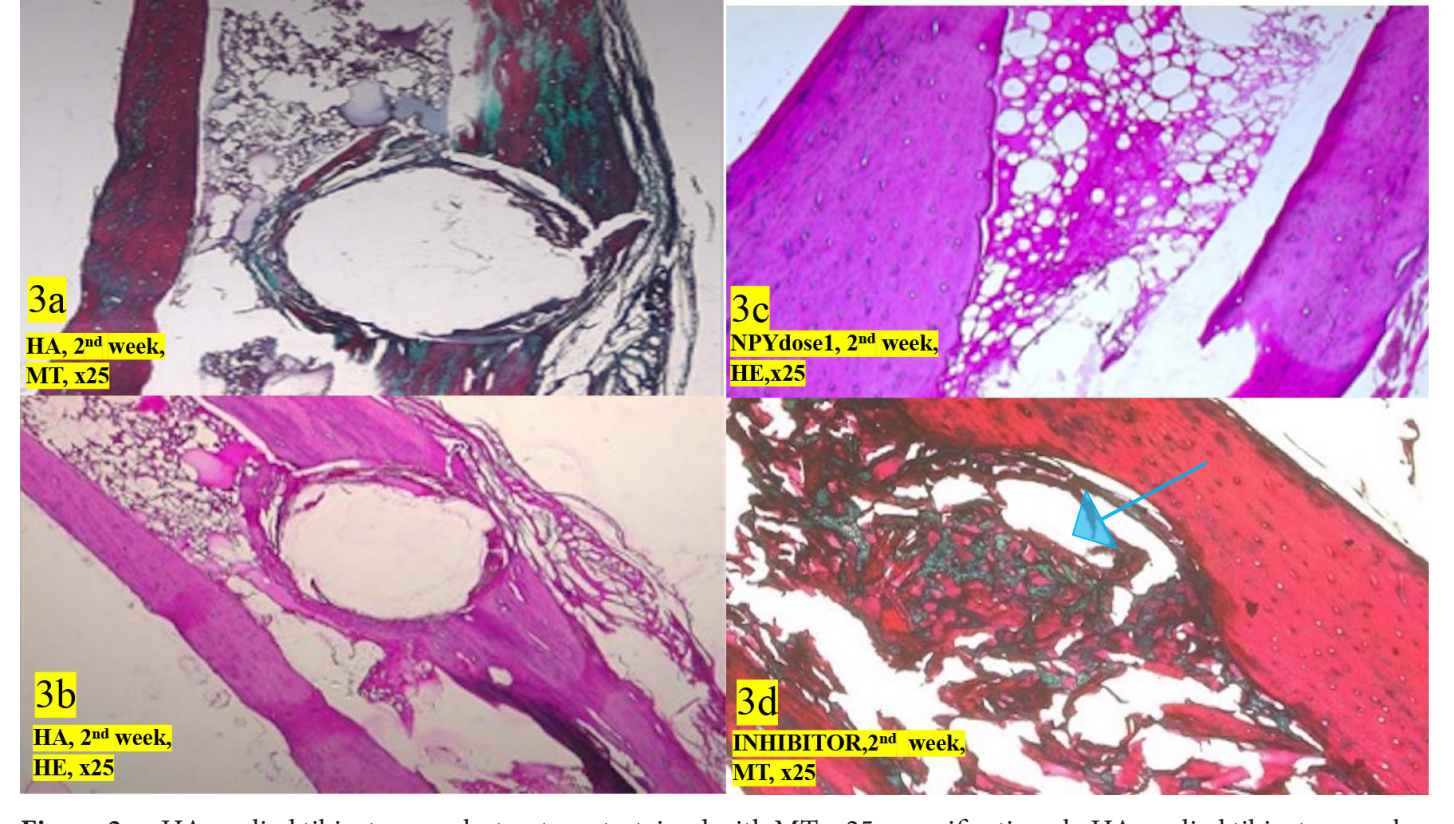
a: HA applied tibia, two weeks treatment, stained with MT, ×25 magnification; b: HA applied tibia, two weeks treatment, stained with HE, ×25 magnification; c: NPY dose1 applied tibia, two weeks treatment, stained with HE, ×50 magnification; d: NPY1 receptor antagonist applied tibia, two weeks treatment, stained with Masson trichrome technique, ×25 magnification. The arrow shows the green stained new bone formation.

Histological analysis revealed that on the fourteenth day (week two), HA only (Figures 3a and 3b) and NPY (Figure 3c) applied groups did not reveal any significant difference in terms of bone healing and tissue response to the system. Nevertheless, the NPY1 receptor inhibitor application revealed young spongiosis bone trabeculae in the borderline of cortical bone as a thin green layer (shown in blue arrow) when stained with Masson trichrome technique (Figure 3d). 

## 4. Discussion

The annual number of fractures in the European Union was 3.5 million in 2010, and this will be expected to reach 4.5 million in 2025 [27]. In the USA alone, trauma, osteoporosis, and genetic diseases cause approximately 6 million fractures annually [28]. However, between 5% and 10% of all fractures result in delayed union or nonunion depending on the age, smoking habit, and diabetes history of the patient [28]. Some fractures will not even heal without further intervention [29]. Due to other systemic diseases that patient already bears or blood circulation at particular bone fractures sites, pharmacological intervention may not always be possible systemically. Therefore, the need for new therapeutic strategies to improve bone regeneration is apparent [30]. One promising strategy is focusing on small molecules applicable in biodegradable matrices that will create a local bone formation, without inducing immune reactions but show stable bioactivity [31]. In agreement, our study investigated the effects of NPY and NPY1 antagonist in HA matrix as possible modulators of local bone formation that will minimize off-target tissue effects of systemic treatments but create bone mass change at the application site. 

Skeletal sympathetic and sensorial nerve fibers are directly involved in the control of bone turnover through neurotransmitter receptors expressed by bone cells [12]. However, significant deficits do exist related to the local actions of the nervous system in bone homeostasis [32], as discussed in the following lines. First of all, NPY signaling in the central nervous system has been shown to contribute to a broad range of physiological processes, including control of feeding behavior, energy homeostasis, vascular and immune function, pain, and stress coping [33] mainly through Y2 receptors. In the peripheral bones, NPY seems to exert its actions through Y1 receptors [34] and is expressed by nonneuronal cells such as bone cells (osteoblasts and osteocytes), bone marrow cells, and endothelial cells [35]. Therefore, Y1 receptors have become a drug target for researchers. In a study by Sousa et al., the ad-libitum application of NPY Y1 receptor antagonist was shown to increase bone mass without serious side effects in mice [34]. This was concordant with previous findings in the literature reviewed by Motyl et al. [36] indicating that mice with a global deletion of the Y1 receptor had high BMD due to elevated osteoblast activity and shown that peripheral NPY system was also a key player in bone metabolism [37]. Besides, osteoblast-specific deletion of the Y1 receptor, but not hypothalamic-specific deletion, also increases osteoblast activity and bone volume, and deletion of Y1 receptors from bone marrow stromal cells (BMSCs) promotes osteoblast differentiation [38]. Consistent with this, it was also shown that the release of NPY from nerves residing in the peripheral skeleton inhibits bone formation through Y1 receptor-mediated inhibition of cAMP and ERK pathways [39]. Also, germline and osteoblast specific Y1R deletion were shown to promote an increase in bone mass [40], and the same effect was obtained after ad libitum pharmacological blockage of NPY Y1 receptor [21]. Finally, studies with a knock-out mice model of NPY indicated that these mice had significantly increased bone mass in association with enhanced osteoblast activity. Global knock-out of NPY results in a smaller femoral cortical cross-sectional area (–12%) and reduced bone strength (–18%) in male mice [17]. However, despite both sexes presenting with increased adiposity, female mice had no alterations in bone mass, suggesting that NPY may have sex-specific effects on bone. The differential impact of NPY deletion in cortical and cancellous compartments along with differences in phenotypes between in vitro and in vivo highlights the complex nature of NPY signaling [17]. Despite all this information, no previous studies tried combining the NPY signaling pathway's bone modulation potential and HA to obtain a local bone mass change effect.

Hyaluronic acid is a glycosaminoglycan that is present in all vertebrates. It is well tolerated and has excellent biomechanical properties. Hyaluronan has been reported as a potential agent for bone regeneration and a drug delivery system in bone healing [41,42], especially in hydrogel form, when functionalized with growth factors [43]. 

In our study, the HA system was biocompatible with the neighboring tissue as a drug carrier. HA seemed to exist inside the medullary cavity near the injection zone, seemed to withstand for some time, seemed to resorb gradually within three weeks, and displaced itself with the new bone tissue and new medullary elements. Also, at the end of week two, histological findings displayed that new bone formation occurred in the intramedullary area of the bone when NPY plus inhibitor was applied. This confirms the gradually resorbing nature as a drug carrier since no immune reaction was observed. According to our limited knowledge, this is the first-time usage of HA in local delivery of NPY or NPY antagonists in the literature.

Our results indicate that there existed a time-dependent, consistent, and significant gain in the BMD of tibiae treated with NPY1 inhibitor compared to NPY only or HA only (carrier only) applied tibiae. We can infer from this result that NPY has BMD reducing (osteoclastic) effect by some intrinsic mechanisms involving the NPY1 receptor. This effect is almost dose-dependent, and the application of NPY1 antagonists by dissolving them in HA causes a significant increase in bone mass. This result was concordant with other studies such as Sousa et al.'s [34] of which indicated that pharmacological ad libitum NPY1 antagonist (BIBO3304) treatment in wild type male mice ( C57/BL6 mice) causes an increase in bone mass over an 8-week treatment period. However, our study showed that with the utilization of hyaluronic acid (HA), the NPY system could be applied as a therapeutic strategy locally for drug applications. Limitations of our study were a low number of rats to experiment and a relatively short time of treatment periods. 

Our findings will hopefully lead to a further understanding of complex neuromediator mediated neural network and neuromediator signaling pathways in the bone. Further studies are needed with longer treatment times with more animals to delineate complex NPY-bone regulation network.

## Acknowledgments

This work was supported by Middle East Technical University (Ankara, Turkey) project fund BAP-2003-07-02-000-32. We would like to thank to Professor Dr. Alpaslan Şenköylü for all his support, Prof. Dr. Ruşen Geçit for sharing their facilities, Prof. Dr. Aykut Özkul, Ankara University, for helping and checking preparations of stock solutions, Prof. Dr. Kamil Can Akçalı for support in finding animals, and Prof Dr. Serhat Akın for the QCT scans.

## References

[ref1] (2011). Disorders of bone remodeling. Annuual Review of Pathology.

[ref2] (2020). Mechanotransduction in osteogenesis. Bone Joint Research.

[ref3] (2018). Long-term consequences of traumatic brain injury in bone metabolism. Frontiers in Neurology.

[ref4] (1963). The nature of bone changes associated with nerve injuries and disease. Journal of Bone and Joint Surgery.

[ref5] (1965). Lower extremity fractures in patients with spinal-cord injury. The Journal of Bone and Joint Surgery.

[ref6] (1963). Pathological ossification in traumatic paraplegia. The Journal of Bone and Joint Surgery.

[ref7] (2002). Adrenergic regulation of bone metabolism: possible involvement of sympathetic innervation of osteoblastic and osteoclastic cells. Microscopic Research Techniques.

[ref8] (2002). Expression and regulatory role of receptors for vasoactive intestinal peptide in bone cells. Microscopic Research Techniques.

[ref9] (2013). Neuropeptide Y receptors: how to get subtype selectivity. Frontiers in Endocrinology (Lausanne).

[ref11] (2012). Central and peripheral mechanisms of the NPY system in the regulation of bone and adipose tissue. Bone.

[ref12] (2019). Roles of neuropeptide Y in neurodegenerative and neuroimmune diseases. Frontiers in Neuroscience.

[ref13] (2018). Impact of the autonomic nervous system on the skeleton. Physiological Reviews.

[ref14] (2018). A promising therapeutic target for metabolic diseases: neuropeptide Y receptors in humans. Cellular Physiology and Biochemistry.

[ref15] (2009). NPY revealed as a critical modulator of osteoblast function in vitro: new insights into the role of Y1 and Y2 receptors. Journal of Cell Biochemistry.

[ref16] (1989). Fixation and demineralization of bone tissue for immunohistochemical staining of neuropeptides. Calcified Tissue International.

[ref17] (2004). Emerging neuroskeletal signalling pathways: a review. FEBS Letters.

[ref18] (2019). Skeletal phenotype of the neuropeptide Y knockout mouse. Neuropeptides.

[ref19] (2016). Bone injury and repair trigger central and peripheral NPY neuronal pathways. PLoS One.

[ref20] (2009). NPY signalling pathway in bone homeostasis: Y1 receptor as a potential drug target. Current Drug Targets.

[ref21] (2009). Neuropeptides and their receptors: innovative science providing novel therapeutic targets. British Journal of Pharmacology.

[ref22] (2012). Neuropeptide Y Y1 receptor antagonism increases bone mass in mice. Bone.

[ref23] (2019). Skeletal phenotype of the neuropeptide Y knockout mouse. Neuropeptides.

[ref24] (2012). Bone-specific overexpression of NPY modulates osteogenesis. Journal of Musculoskeletal Neuronal Interactions.

[ref25] (2020). The application of hyaluronic acid in bone regeneration. International Journal of Biological Macromolecules.

[ref26] (2003). Target-specific neuropeptide Y-ergic synaptic inhibition and its network consequences within the mammalian thalamus. Journal of Neuroscience.

[ref27] (1998). The NPY Y1 receptor antagonist BIBP 3226 blocks NPY induced feeding via a non-specific mechanism. Regulatory Peptides.

[ref28] (2013). Osteoporosis in the European Union: medical management, epidemiology and economic burden. Archives of Osteoporos.

[ref29] (2008). Critical analysis of the evidence for current technologies in bone-healing and repair. The Journal of Bone and Joint Surgery. American Volume.

[ref30] (2010). Testing the critical size in calvarial bone defects: revisiting the concept of a critical-size defect. Plastic and Reconstructive Surgery.

[ref31] (2019). Innovative biomaterials for bone regrowth. International Journal of Molecular Science.

[ref32] (2020). Adjuvant drug-assisted bone healing: Part I – Modulation of inflammation. Clinical Hemorheology and Microcirculation.

[ref33] (2019). Bone and the Brain. Basic and Applied Bone Biology.

[ref34] (2012). NPY and stress 30 years later: the peripheral view. Cellular and Molecular Neurobiology.

[ref35] (2012). Neuropeptide Y Y1 receptor antagonism increases bone mass in mice. Bone.

[ref36] (2010). Neuropeptide Y expression. FEBS Journal.

[ref37] (2019). Bone and the Brain. Basic and Applied Bone Biology.

[ref38] (2009). Neuropeptide Y knockout mice reveal a central role of NPY in the coordination of bone mass to body weight. PLoS One.

[ref39] (2011). Osteoblast specific Y1 receptor deletion enhances bone mass. Bone.

[ref40] (2017). NPY and CGRP inhibitor influence on ERK pathway and macrophage aggregation during fracture healing. Cell Physiological Biochemistry.

[ref41] (2007). Novel role of Y1 receptors in the coordinated regulation of bone and energy homeostasis. Journal of Biological Chemistry.

[ref42] (2014). Artificial matrices with high-sulfated glycosaminoglycans and collagen are anti-inflammatory and pro-osteogenic for human mesenchymal stromal cells. Journal of Cellular Biochemistry.

[ref43] (2017). Collagen/glycosaminoglycan coatings enhance new bone formation in a critical size bone defect — a pilot study in rats. Materials Science and Engineering: C.

[ref44] (2014). Photo-cured hyaluronic acid-based hydrogels containing growth and differentiation factor 5 (GDF-5) for bone tissue regeneration. Bone.

